# Covid and Public Funds: More Opportunities for a Misuse? The Case of the Intermediate Governments of Galicia

**DOI:** 10.1007/s11115-022-00638-5

**Published:** 2022-06-18

**Authors:** Bruno Blanco-Varela, María Quintas-Pérez, María Carmen Sánchez-Carreira, Paulo Jorge Reis Mourão

**Affiliations:** 1 Departament of Applied Economics, Faculty of Economics and Business, Universidade de Santiago de Compostela, Avda. do Burgo, s/n, 15782 Santiago de Compostela, Spain; 2 Departament of General Public Law and Research Centre for Global Governance, Faculty of Law, University of Salamanca, Campus Miguel de Unamuno, Avda. Francisco Tomás y Valiente, s/n, 37007 Salamanca, Spain; 3 Departament of Applied Economics, ICEDE Group, Faculty of Economics, CRETUS Institute, Universidade de Santiago de Compostela, Avda. do Burgo, s/n, 15782 Santiago de Compostela, Spain; 4 Department of Economics & NIPE, Economics & Management School, University of Minho, 4700 Braga, Portugal

**Keywords:** Corruption risk, Indicator, Local government, Local public procurement, Minor contract, Covid

## Abstract

Corruption and inefficiency of public funds pose a risk in public administrations. This paper analyses the corruption risk at the local level by analysing indicators of public procurement contracts in four deputations of Galicia (Spain). In addition, the pandemic has created opportunities to increase this risk and the misuse of public funds given the need to act quickly. Therefore, the study analyses whether the Covid crisis led to significant changes in expenditure in the four deputations and whether it involves a higher use of minor contracts, an award procedure without publicity or bidding, which has been found as increasing corruption risk.

## Introduction

The effects of corruption are considered among the most damaging at the local government, which is the closest level of government to citizens (González, 2006). Corruption risk at the local level may be more important than at the regional and national level (European Commission, 2014; Habibov et al., 2019). The corruption risk may be due to several factors: organisational weakness of the local administration, the pre-existence of clientelist networks (power structure at the local level), a government with a high concentration of power in few persons or only one, more discretionary power, ineffective control mechanisms and less media attention (Kwon, 2014; Loftis, 2014; Jiménez, 2016; Slijepčević et al., 2020).

The emergency may have led to many policy gaps, being an ideal breeding ground for the misuse of public funds. This misuse leads to lower quality of public services, lower economic growth, inefficiency in the decision processes, and citizens distrust (d'Agostino et al., 2016). Corruption can take the form of patronage, nepotism, clientelism, preferential allocation of public procurement, abuse of power, or conflict of interest (Slijepčević et al., 2020). It has been found as frequent in certain public procurement and public service deliveries (Tromme & Volintiru, 2018).

Recent literature highlights the corruption risk in local public administrations, as well as the link between corruption risk and pandemic. This corruption risk is extended by the general competences, the unawareness, and the lack of accountability (Jiménez, 2011; FHD, 2016). When there is an emergency, governments must act quickly; therefore, risk increases. In this sense, it can lead to uncoordinated procurement and increased opportunities for fraud or integrity failures (Schultz & Søreide, 2008; OECD, 2021). Rose-Ackerman (2021) notes that lack of time undermines the quality and transparency of the procurement process.

The aim of this paper is to analyse the effect of the pandemic on increasing the corruption risk in local public procurement. It examines minor public procurement contracts carried out during the pandemic in the Spanish local public administration. Minor contracts are a specific type of public procurement highly exposed to corruption practices. Specifically, this work focuses on deputations, the provincial governments (NUTS 3) in Spain, analysing their corruption risk. This topic has hardly been approached in literature; however, there is a growing interest in this issue, given the extraordinary situation arising from the Covid 19 pandemic. It affects procurement and economic procedures, as well as the management of a substantially increased volume of public funds. The results contribute to the debate on how the pandemic led to an increased corruption risk in local public procurement.

The analysis of Spanish local public administration represents a worthy case study for diverse reasons. Firstly, Spain has been severely affected by the pandemic and forced to give a quick response. Secondly, the analysis of Spanish local government is interesting because of the high level of decentralisation and asymmetric regionalism. The multilevel organization of the Spanish public administration involves challenges for governance and the need for effective coordination to manage the emergency. Thirdly, the Spanish intermediate governments (deputations) face difficulties considering the dimension of accountability because they are entities focused on expenditure and characterised by the lack of own revenue-raising capacity. Fourthly, there is a diversity of responses to the Covid emergency at the local level, in particular, in the deputations, derived from the flexibility in the execution of its competences. Although deputations have limited competences, these competences are vague and can be executed with a certain degree of discretion. All this makes it interesting to study how they managed the emergency resulting from covid.

Methodologically, this paper combines a literature review and legislation analysis with non-parametric data analysis of minor public procurement contracts. Changes in minor public procurement contracts in the four deputations derived from Covid are specifically analysed. Four deputations from the same region (Galicia) are selected because they show several patterns of management and different responses to the emergency. The study focuses on the awarding procedures and awarded companies in the period 2018–2021.

This paper is organised into five sections, apart from this introduction. The second section approaches the theoretical framework about corruption risk in the Covid context in public procurement. The third section describes the role of the deputations, their competences, and the minor contract as recurrent expenditure. The fourth section analyses the evolution of their spending in the pandemic, as well as the corruption risk that they face. The fifth section examines the corruption risk through indicators of local public procurement focusing on the effect of pandemic, after explaining the methodology and the analysed data. Finally, conclusions and recommendations are presented.

## The Theoretical Framework: Public Procurement, Corruption Risk, and Covid

Public procurement is one of the government tools available for achieving public goals, accounting for around 10% of Gross Domestic Product in Spain (OECD, 2021). It has been found as highly exposed to risks of irregular management and corruption (Auriol, 2006; Transparencia Internacional, 2018; OECD, 2021). The consequences of irregularities in this area involve the inefficient use of public funds, competition distortion that leads to higher prices, and managers distrust (Gimeno, 2010). In this regard, the European Council has pointed out in its Decision 2017/984 of August 8 2016 that there are divergences in the execution of public contracts in Spain (Council of the European Union, 2016). In addition, the control mechanisms are insufficient, which hinders the correct and uniform application of public procurement legislation. The decision also highlights the low rate of tenders publication, as well as an overuse of the negotiated procedure without prior publication compared to other Member States. This often results in direct awards, which can lead to increased spending by public administrations.

Direct award and, specifically, minor contracts are one of the most relevant types of public procurement. This fact is not unique to Spain, as the direct award is a method also abused in the EU Member States. Thus, for example, 90.2% of the number of contracts are directly awarded in Portugal, representing 47.9% of all public procurement expenditure (Telles, 2017). The features of this procedure present several risks for corruption (see “Deputations: A Discussion Focused on Governance Issues” section).

In addition to these usual risks, the exceptional situation resulting from the pandemic could have created more opportunities for the misuse of public funds (Anessi-Pessina et al., 2020; Utrilla, 2021). Thus, several factors have impacted on corruption derived from the Covid emergency (Anessi-Pessina, et al., 2020): increased level of resources to address the emergency, increased discretionary of processes, loosening of transparency and accountability mechanisms, or reduced monitoring measures. In addition, urgency is often considered an element of corruption risk in public procedures. In this sense, some literature highlights the risk of inefficient procurement in pandemic times because it has driven governments to increase spending substantially and rapidly (Gallego et al., 2021). These situations of immediate increases in public spending could lead to additional corruption risk, such as Centorrino and Ofria (2003) suggest. In addition, Transparency International (2021) has warned that Covid may have been hampering the fight against corruption in some countries. It also noticed that many governments have loosened or neglected their procurement processes (including the EU regulation), prioritising speed over transparency.

Thus, the pandemic may have potentially exacerbated corruption. In this sense, several investigations have highlighted that in pandemic or emergency situations anti-corruption tools play a key role (Mrčela, 2020; Transparency International, 2020). In this context, it has been crucial that governments follow the principles of accountability and transparency (Mrčela, 2021). Transparency is essential to ensure compliance with legal procurement procedures, promote competition and allow for the control of bias in the contract awarding. Anessi-Pessina et al. (2020) and Mugellini et al. (2021) argue that a new framework is needed to effectively address corruption and abuses even more during emergencies. In this sense, citizen participation is crucial to ensure good governance, but also the deployment of mechanisms such as effective audits, accountability, and transparency.

## Deputations: A Discussion Focused on Governance Issues

This section tackles the deputations as an intermediate local government level in Spain and their competences. It focuses on the legal aspects and on the governance issues related to these deputations. In addition, the theoretical framework of minor contract procedures is considered. Thus, this section provides background to the specific case study of Galician deputations, taking into account the local entity under analysis and a specific expenditure procedure.

### Nominated Governments: Is Citizen Governance Possible?

According to the Spanish Constitution, the State is organized territorially into three main levels: Autonomous Communities (17), provinces (50), and municipalities (8,131). In addition, the deputation is the government and administrative body of a province, which is composed of municipalities. Each of these types possesses autonomy for the management of their interests. Most of the municipalities (91%) have less than 10,000 inhabitants. This hinders the implementation of certain public policies because they require a broader territorial scope, given the lower costs of the aggregated provision or the insufficient resources to undertake them by a single municipality (Jiménez, 2011).

Intermediate local governments, such as the deputations (NUTS 3), play a key role in this structure. Their role is to enhance cooperation to municipalities, especially those with less economic and management capacity, and to ensure the provision of the minimum services entrusted to them.

The representatives of the intermediate government (deputies) in Spain are not directly elected by the citizens, but by the representatives of the municipalities that compose the province (councillors). Thus, citizens do not have a candidate to vote for but elect the councillors of the municipalities, who appoint the provincial deputies among themselves. This system means that accountability to the electorate, who often do not even know their representatives, is neither operative nor effective (FHD, 2016). This lack of accountability has a direct impact on the corruption risk. This risk may increase in the context of an emergency, as already explained in “The Theoretical Framework: Public Procurement, Corruption Risk, and Covid” section.

### Minor Contracts in the Deputations: The Gold Medal of Fast-Track Procedures

The deputations can procure through several types of contracts according to different criteria, such as the object (works, supplies, services), the procedure (ordinary, urgent, or emergency) or the award method (open or negotiated). The rules to be followed for publication, awarding, or deadlines, among other aspects, change from one typology to another.

Public procurement must comply with the principles of free access to tenders, publicity and transparency of the procedures, and non-discrimination and equality of treatment among suppliers. The law regulates a series of award procedures that aim to fulfil these objectives and avoid corruption. The open procedure and the restricted procedure are set as the ordinary and preferential procedures. Moreover, the law indicates other procedures restricted to certain (extraordinary) cases, such as the negotiated procedure, competitive dialogue, the innovation partnership, or minor contracts.

Minor contracts are intended to allow the Administration to procure products and services on a lower scale in a more agile way. The estimated value of this contract should be less than 40,000 euros for works, or 15,000 euros for supply or service, while there is no quantitative limit for the access to databases and subscriptions to publications. The duration may neither exceed one year nor be extended.

In this type of contract, given its relatively small scale, a large part of the formalities required in overall award procedures can be neglected. Thus, the expedient in the contracting body merely requires reporting the need for the contract and that it is not unduly splitting, approving the expenditure, and incorporating the corresponding invoice, as well as the budget in the case of works contracts.

Carrodeguas (2018) considers that requiring the report to demonstrate that it is not unduly splitting shows the legislator distrust of the public manager. This is due to the common use of splitting minor contracts to circumvent ordinary procedures. Indeed, Baena (2018) underlines that one of the usual risks in the contract preparation phase is unduly limiting competition. This situation happens when the description of the object of the contract is tailored to a particular economic operator. Thus, the object of the contract is artificially split in such a way that the estimated value allows the selection of procedures with lower processing requirements. This is also considered a risk by the Consello de Contas (2018).

Moreover, minor contracts can be directly awarded, being one of the most widely used forms of procurement in Spain, rather than an exceptional procedure. A report of the Consello de Contas (2021) notes that the most widely used award procedure by all the local entities in Galicia (Municipalities and Deputations) in 2018 was the minor contract. This type accounted for 89% of all contracts and 14% of the amount awarded.

The direct award may be effective and necessary on certain occasions. Opposite to this simplified procedure of minor contracts, the open procedure means that the tender announcement is published in the contracting entity profile, and anyone can submit a proposal. It is neither a simple nor a short procedure, as it lasts at least six months, but it ensures transparency, and objectivity (Royo, 2018).

Nevertheless, the problem arises when their use is abusive and sometimes self-interested. Direct award does not require competition or advertising. This implies a lack of transparency, which can favour corruption and economic inefficiency (Carrodeguas, 2018). Thus, the use of handpicked contracts goes against the general principles of public procurement, such as publicity, free access to tenders, free competition, and the selection of the most advantageous economic offer.

Direct, non-transparent, and unpublicised awards, such as minor contracts, can encourage the misuse of public funds and lead to economic inefficiency. The report of CNMC (2019) assessed the impact of using the most competitive procurement procedures on economic efficiency, at the level of the Central Administration and without including minor contracts. When the open procedure is used instead of the non-open procedure, the administration pays 9.9% less on average. Furthermore, it notes the decrease of contract amounts derived from competitive awards. Thus, the additional participation of one company in the competition process results in a reduction of the average amount of 2.1%. This impact has been found more significant for works contracts than for service and supply contracts.

As evidence of these problems and misuse of public funds, it is common to find contracts just at the limit of the thresholds. It is noteworthy that when the award thresholds for minor contracts decreased in March 2018 (from €18,000 to €15,000 and from €50,000 to €40,000, respectively), the amount of many of these supplies and services contracts also changed. It raises the crucial question of whether there was overpaying or if there are suppliers willing to accept lower payments to maintain contracts, as Belmonte and Cabo (2020a) pose.

Moreover, actions of a necessary, repeated, and expected nature or the performance of services of a similar nature that respond to a single purpose are separately awarded. Indeed, multiple contracts are awarded to the same supplier (Belmonte & Cabo, 2020a). This may be due to several reasons, such as inadequate planning of the actions required to meet public needs, trust on the supplier, agility, or security in the service quality. Nevertheless, audit bodies often conclude that similar services provided by the same contractor, especially if the award is close in time, constitute a splitting of a contract or its object (JCCAA, 2009). This is undertaken to circumvent the legal award procedures and advertising requirements (Tribunal de Cuentas, 2020).

## Pandemic Deputations: Effect on Expenditures

This section introduces and discusses the quantitative data of Galician deputations. It also analyses the evolution of public budgets over the period 2010–2021 and how the volume of spending has reacted to the pandemic. The MAD methodology is used to study whether structural changes have occurred during the pandemic.

### Growing Budgets: A Long-Distance Race

**Fig. 1:**
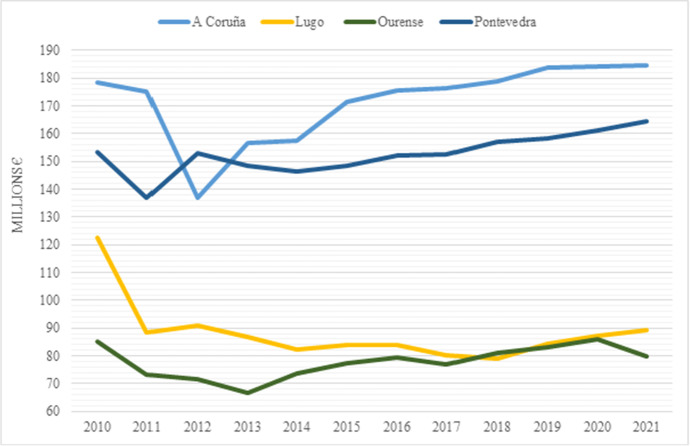
Evolution of expenditure in the Galician deputations in 2010–2021. Source: Own elaboration based on Ministerio de Hacienda y Función Pública (2010-2021)

This section analyses the expenditure structure and the budgetary effect of pandemic for these four Galician intermediate governments. One of the effects of the pandemic is the increase in public expenditures. In this regard, it is important to present the magnitude of the resources managed by the deputations in pandemic times and how the public administration has performed in the emergency. For this reason, Fig. 1 depicts the budget executed by the four deputations, which has slightly increased in recent years, except for Deputation of Ourense, which shows a small decrease in 2021.

The evolution of the deputations budgets in the period analysed is heterogeneous, as Table 1 shows. Thus, the budget decreases in Deputations of Lugo and Ourense, while it increases in A Coruña and Pontevedra. In addition, there is disparity in the variation rates among the four deputations. Focusing on the latest period 2015–2021, which coincides with economic recovery, the four deputations recorded budget growth rates. This growth trend is maintained in the context of the pandemic and even accelerates in three deputations. This can be noted when comparing the cumulative annual growth rate during 2015–2021 with that of 2019–2020. It should be highlighted that in the pandemic shock, the budget in Lugo and Ourense shows the highest growth.

**Table 1:** Evolution of budgets in Galician deputations 2010–2021

Period	Variation rates	A Coruña	Lugo	Ourense	Pontevedra
2010–2021	Variation rate	3.35%	-27.20%	-6.17%	7.43%
	Cumulative rate	0.28%	-2.61%	-0.53%	0.60%
2019–2020	Variation rate	0.22%	3.49%	3.69%	1.88%
2015–2021	Variation rate	7.68%	6.33%	3.24%	10.81 Lede
	Cumulative rate	1.06%	0.88%	0.46%	1.48%

Source: Own elaboration based on Ministerio de Hacienda y Función Pública (2010-2021)

The expenditure areas of the deputations are diverse, and they can be grouped into six main headings: basic public services, general interventions, economic initiatives, social protection and promotion actions, production of public goods of a preferential character, and public debt. Another relevant characteristic is the vagueness of the competences of deputations, which allows spending with certain degree of discretion within the framework of their competences. Thus, each deputation can choose how to distribute the resources and their investments. The intermediate governments of Ourense and Lugo spend a higher relative budget share in basic public services in 2020. It should be noted that both these provinces show a high percentage of less dense municipalities. The deputations of A Coruña and Ourense have mainly spent on economic initiatives in 2020, while the Deputation of Pontevedra spent relevant values in general intervention, which encompasses control and audit functions.

### Data and Methodology

To identify structural changes further away from the central measures, either by the Covid effect (years 2020 and 2021) or by observations that stand out for another cause, a 'Median Absolute Deviation' (MAD) Analysis is performed. MAD Analysis, proposed by Leys et al. (2013), tests whether the values in observation are outside the range of Sample Median ± Median Absolute Deviation interval. Thus, by MAD Analysis, and assuming a normal distribution in the variables, the values that tend to fall in the 15% of observations with smallest values and in the 15% of observations with largest values are signalled as 'outliers'. It should be clarified that the outliers are detected by looking at the entire sample under scrutiny. This is a non-parametric analysis suggested by several authors (Dwivedi et al., 2017; Noether, 1987) given the limitation of certain databases similar to the data used in this research (small number of time observations).

### Results

A relevant aspect regarding the analysis of the budgets is to study whether changes occurred to adapt to the Covid context. To this end, Table 2 shows the differences between the planned and the actually executed budgets in 2020, as well as the comparison with 2019, the year prior to the pandemic. The items that changed the most before and during the pandemic are related to basic public services and general interventions.

**Table 2:** Variation rate of planned and executed budgets in Galician deputations 2019–2020

Deputation	Year	Public Debt	Basic public services	Social protection and promotion actions	Production of public goods of a preferential character	Economic initiatives	General interventions	Total Expenditure
A Coruña	2020	-	83.9%***	4.0%	-11.1%	33.2%	-43.5%	7.9%
	2019	0.0%	90.2%***	-13.4%	-4.1%	11.3%	-31.6%	4.0%
Lugo	2020	-3.8%	57.7%	-10.7%	3.5%	12.8%	-53.6%***	-2.8%
	2019	-31.8%	56.4%	-6.3%	3.3%	28.0%	-48.5%	6.1%
Ourense	2020	3.9%	1.4%	48.5%	12.9%	7.6%	-9.4%	8.7%
	2019	-66.7%***	-11.4%	3.8%	-2.9%	-7.8%	-10.4%	-8.2%
Pontevedra	2020	35.6%	10.5%	5.4%	-38.2%	13.0%	-2.5%	0.1%
	2019	-1.0%	28.3%	-8.2%	-9.7%	22.1%	7.4%	7.7%

***. 5% significance value of being an Outlier

Source: Own elaboration based on Ministerio de Hacienda y Función Pública (2010-2021)

Table 2 depicts that the year 2020 has only shown changes signalled by the MAD Analysis in some items for some deputations: Public Services (+ /Coruña), and General Interventions (-/Lugo). Thus, according to these data it cannot be signalled in a special way the Covid effect for most of the dimensions in Table 2.

For the 2021 budget year, expenditure is still being processed. It should be noted that for the preparation of the 2021 budget, the situation resulting from Covid has already been considered. Table 3 presents the shares of the expenditure items for the four deputations, showing that different deputations have different outliers in 2021: Lugo and Pontevedra in general interventions (40.1% and 51%, respectively) and Ourense in basic public services (26%).

**Table 3:** Planned budget in deputation of Galicia by expenditure areas in 2021

Deputations	Public Debt	Basic public services	Social protection and promotion actions	Production of public goods of a preferential character	Economic initiatives	General interventions
A Coruña	0.0%	1.6%	29.8%***	14.7%	26.1%	27.8%
Lugo	1.9%	7.6%	15.7%	13.7%	21.0%	40.1%***
Ourense	1.0%	26.0%***	7.3%	11.8%	30.6%	23.4%
Pontevedra	0.5%	3.7%	13.4%	14.9%	16.4%	51.0%***

***. 5% significance value of being an Outlier (MAD)

Source: Own elaboration based on Ministerio de Hacienda y Función Pública (2010-2021)

The analysis of these data suggests that part of the spending of Galician deputations reports in 2020 a statistically significant increase compared with previous years. However, it was not found evidence that Galician deputations have substantially modified their budget distribution.

Anyway, rather than examining significant changes in spending amount or distribution, it is more relevant to analyse how spending is executed and how procedures are being managed. In this sense, spending can be allocated through faster procedures and under less control in times of pandemic. This fact entailed higher opportunities for corruption risk, as previous literature highlighted (Centorrino & Ofria, 2003; Gallego et al., 2021).

## A Step Further in Budgets: Assessing Corruption Risk in Local Public Procurement during Covid

This section approaches the increase of corruption risk in local public procurement during pandemic. To this end, the analysis focuses on minor contracts in the period 2018–2021 in four Spanish deputations. Minor contracts are a usual type of contract in local procurement, which entails high corruption risk due to its characteristics and procedures (see “Deputations: A Discussion Focused on Governance Issues” section). Next, the used data, the methodology, and the results are presented.

### Data and Methodology

Before examining corruption risk, the sources of information on local public procurement and their limitations are approached.

The transparency in public procurement is ruled by Law 19/2013. Thus, it is required the publication of information in a clear, structured, and understandable way for involved stakeholders, preferably in reusable formats. In the case of minor contracts, the information must be published at least quarterly and include the following aspects: object, duration, amount allocated, and contractors awarded. Despite this regulation, in many cases, the legal conditions are not met; then, adequate public access is not allowed.

There are different sources of information on public procurement in Spanish public administrations. Local government contracting bodies must choose whether to publish their contracting profiles on the general Public Sector Procurement Platform (Plataforma de Contratación del Sector Público, PCSP) at the national level, the information service established by each region, or their local transparency web portal. In this sense, the lack of homogeneous data about minor contracts, and reusable information publicly available about the local public procurement hinders transparency and control. This is a factor that can affect accountability and the fight against corruption (Jiménez, 2016; CNMC, 2019). In this regard, JCCE (2019) establishes that reusable formats are mandatory in the contracts advertising. Nevertheless, it is widespread practice to publish a PDF file or a spreadsheet with the contracts concluded in shorter periods.

The information for Deputation of Ourense was obtained from the Public Procurement Platform. However, the information provided on this portal for the other three deputations was not complete. As an illustrative example, the Deputation of Lugo had only 11 minor contracts registered from 2018 to September 2021, while 3,407 contracts were identified on their transparency portal. Given the lack of systematised information, for this analysis an own database is developed, including information from both sources: national and local portal webs. All this information is refined, checking for homogeneity and eliminating duplicities.

As a first step to assessing corruption risk in local public procurement and the effect of pandemic, this section focuses on local minor contracts. This issue is relevant for corruption risk, given the significant percentage of minor contracts in the total value of contracts, the features of these contracts (see Sect. 3), as well as the widespread trend to abuse them.

Literature suggests that there is an association between corruption risk and the concentration level of suppliers of goods and services regarding the provision to the state (Heeks, 2011; Open Government Partnership, 2020; Søreide, 2002; Telles, 2017). Thus, it is expected that State figures (Central, Regional, or Local), which are more dependent on certain suppliers, tend to be more likely to generate ‘political rents’ (which are excessive amounts of expenditure in relation to the derived social utility). These ‘political rents’ tend to generate, in turn, greater corruption risk. Equation 1 synthesizes the foundational model of this research.

1 $$\mathrm{Corruption}\;\mathrm{Risk}=\mathrm f\lbrack\mathrm{Political}\;\mathrm{rents}=\mathrm g(\mathrm{Concentration}\;\mathrm{of}\;\mathrm{Public}\;\mathrm{Procurement}\;\mathrm{Contracts})\rbrack$$

For this reason, the concentration of suppliers in minor contracts can be considered an appropriate proxy of local risk corruption. Thus, the fact that the concentration of suppliers increases both in the number of contracts and amount, involves a higher corruption risk.

It should be noted that if there are no judicially confirmed values, these indications of corruption are just working on as a probable risk. A comparable image is that two people walking in the street in different places have different risk of getting wet depending on the rainfall in each place; however, it is not deterministic that the person walking in the street in the rainy place will get wetter than the person walking in the street in the place without rain. Therefore, because there is a greater corruption risk according to certain indicators, it cannot be deterministically ensured that there is more corruption practised in those places, even though this risk is more significant.

Despite concentration is a single dimension, diverse indicators regarding minor contracts are considered in this analysis. Robust indicators for assessing corruption risk require a methodology that allows to discuss observations on several variables. This procedure is followed by different international indicators, such as the Corruption Perceptions Index or the Corruption Watch. Therefore, a non-parametric analysis of the following indicators is also carried out:

- % MC/TB (where MC, minor contract expenditure; TB, total budget);
- Total amount awarded;
- % contracts awarded to the 10 companies with the most contracts out of the total number of companies;
- Total amount awarded to the 10 companies with the highest number of contracts;
- % Amount awarded to the 10 companies with the highest number of contracts awarded;
- Amount to 5 largest awarded companies;
- Amount to 10 largest awarded companies;
- % Amount to 5 largest awarded companies out of total awarded amount;
- % Amount to 10 largest awarded companies out of total awarded amount.

### Results

This section presents the results obtained with the MAD methodology on corruption risk. The methodology is suitable when the number of observations is limited and it is consistent with other relevant literature (Heeks, 2011; Søreide, 2002; Telles, 2017).

Table 4 depicts the relevance of minor contracts in the four deputations in 2018–2021. The Deputation of Ourense shows the largest share of minor contracts during the analysed period. Moreover, minor contracts have increased their relative weight in provincial expenditure, accounting for 10.53% in 2020, while they tend to decrease in the other deputations. Concerning the pandemic situation, the analysis of minor contracts shows an increase both in the number of contracts and the amount awarded for the Deputations of Lugo and Ourense. They are precisely those which used this procedure the most before the pandemic.

**Table 4:** Weight of minor contracts on total deputation budget in 2018–2021

Deputation	Indicator	2018	2019	2020
A Coruña	Total budget (TB)	163,752,015.1	191,233,464.1	199,908,881
	Minor Contract (MC)	980,316.83	572,606.40	824,968.20
	% MC/TB	0.60%	0.30%	0.41%
Lugo	Total budget (TB)	77,262,582.99	89,893,958.33	85,010,995.94
	Minor Contract (MC)	4,637,116.12	2,900,601.34	3,295,860.23
	% MC/TB	6.00%	3.23%	3.88%
Ourense	Total budget (TB)	80,200,512.93	76,711,741.33	94,236,222.22
	Minor Contract (MC)	6,527,509.1	6,631,780.19	9,921,619.29
	% MC/TB	8.14%***	8.65%***	10.53%***
Pontevedra	Total budget (TB)	151,794,828.7	171,280,275.3	161,162,631.9
	Minor Contract (MC)	2,839,096.07	4,561,595.52	2,447,300.54
	% MC/TB	1.87%	2.66%	1.52%

2021 data refer to minor contracts from 1 January 2021 to 30 September 2021. ***. 5% significance value of being an Outlier (MAD)

Source: Own elaboration based on PCSP (2018-2021)

Among direct awards, the most common object of the contract is public works in the case of Ourense, while are services in Lugo (Table 5). It is noteworthy that the use of minor contracts for public works is remarkable in the Deputation of Ourense, even at the national level. Thus, it is one of the administrations with the highest number of presumably split contracts in the period from 01 January 2018 to 31 July 2019 (Belmonte & Cabo, 2020b).

**Table 5:** Distribution of minor contracts by object of contract

Deputation	Object	2018	2019	2020	2021
A Coruña	Public Works	-	13,109.00	52,330.49	-
	Services	18.00	279,039.92	78,718.73	-
	Public Utilities	40,100.55	33,767.17	50,385.12	21,745.46
	Other		63,529.95		
	Total	40,118.55	389,446.04	181,434.34	21,745.46
Lugo	Public Works	603,990.31	364,849.56	583,962.84	436,167.97
	Services	3,019,521.38	1,532,855.70	1,171,406.23	878,155.81
	Public Utilities	1,013,604.43	728,475.88	560,102.52	613,034.92
	Other		274,420.20	980,388.64	349,606.68
	Total	4,637,116.12	2,900,601.34	3,295,860.23	2,276,965.38
Ourense	Public Works	4,696,373.88***	4,417,608.84***	6,599,710.17***	2,784,233.55
	Services	1,506,059.78	1,817,406.29	2,686,123.97	1,874,104.47
	Public Utilities	325,075.44	374,057.12	502,026.48	334,230.85
	Other		22,707.94	133,758.67	83,345.01
	Total	6,527,509.10	6,631,780.19	9,921,619.29	5,075,913.88
Pontevedra	Public Works	369,242.09	997,106.47	639,434.13	399,843.82
	Services	1,980,995.91	2,936,559.46	1,310,547.50	1,050,370.89
	Public Utilities	476,258.07	613,263.23	375,655.97	213,188.87
	Other	12,600	14,666.36	121,662.94	356,449.18
	Total	2,839,096.07	4,561,595.52	2,447,300.54	2,019,852.76

2021 data refer to minor contracts from 1 January 2021 to 30 September 2021. ***. 5% significance value of being an Outlier (MAD)

Source: Own elaboration based on PCSP (2018-2021)

Table 6 shows diverse indicators abovementioned to study the concentration of minor contracts. Among the deputations, the case of Ourense outstands. An increased spending on minor contracts comes with a higher concentration on a small number of suppliers. In other words, the 10 companies that had been awarded the most minor contracts keep their share of public spending relatively stable. When this deputation spends more money, it maintains the share of public spending for those companies that are awarded the most. The 10 companies that handled the most contracts in 2020 took almost a third of the total share of contracts issued.

**Table 6:** Concentration of minor contracts by Deputation 2018–2021

Indicator	A Coruña				Lugo			
	2018	2019	2020	2021	2018	2019	2020	2021
Expenditure minor contracts (MC)	980,316.83	572,606.40	824,968.21	1,251,332.71	4,650,258.82	2,900,601.34	3,295,860.23	2,276,965.38
Total number of minor contracts	143	70	77	216	1485***	846***	684	404
Number of companies with minor contracts	120	62	71	161	670***	436	341	267
Contracts to the 10 companies with the highest number of contracts awarded	27	18	16	47	156***	70	178***	56
% of contracts to the 10 companies with the highest number over the total number of contracts	18.88%	25.71%***	20.78%	21.76%	10.51%	8.27%	26.02%***	13.86%
Total amount awarded to the 10 companies with the largest number of contracts	83,984.11	140,883.97	95,025.83	183,232.86	160,268.83	123,590.51	608,962.24	175,369.69
% Amount awarded to the 10 companies with the highest number of contracts awarded	8.57%	24.60%	11.52%	14.64%	3.45%	4.26%	18.48%	7.70%
Amount to 5 largest awarded companies	157,308.97	145,691.01	166,843.83	149,663.10	322,163.36	222,885.09	638,498.39***	289,967.86
Amount to 10 largest awarded companies	241,733.43	227,016.98	255,516.25	241,140.64	559,011.87	381,679.06	895,107.25***	463,034.71
% Amount to 5 largest awards companies/(MC)	16.05%	25.44%***	20.22%	11.96%	6.93%	7.68%	19.37%	12.73%
% Amount to 10 largest awards companies/(MC)	24.66%	39.65%***	30.97%***	19.27%	12.02%	13.16%	27.16%	20.34%
Indicator	Ourense				Pontevedra			
	2018	2019	2020	2021	2018	2019	2020	2021
Expenditure minor contracts (MC)	6,527,509.10***	6,631,780.19***	9,921,619.29***	5,075,913.88	2,839,096.07	4,561,595.52	2,447,300.54	2,019,852.76
Total number of minor contracts	447	417	624	374	364	515	316	248
Number of companies with minor contracts	247	212	283	217	302	402	267	209
Contracts to the 10 companies with the highest number of contracts awarded	123	142	200	88	38	46	30	33
% of contracts to the 10 companies with the highest number over the total number of contracts	27.52%***	34.05%***	32.05%***	23.53%	10.44%	8.93%	9.49%	13.31%
Total amount awarded to the 10 companies with the largest number of contracts	3,246,084.99***	3,178,479.41***	4,235,689.98***	1,915,835.51	334,580.21	369,054.32	198,852.91	373,802.70
% Amount awarded to the 10 companies with the highest number of contracts awarded	49.73%***	47.93%***	42.69%***	37.74%	11.78%	8.09%	8.13%	18.51%
Amount to 5 largest awarded companies	3,009,183.59***	2,979,250.90	3,912,537.06***	2,009,350.07	247,709.50	279,062.69	214,705.69	397,724.12
Amount to 10 largest awarded companies	3,563,267.88	3,803,174.89	5,179,712.54***	2,633,232.73	431,849.79	499,089.21	403,808.27	548,530.31
% Amount to 5 largest awards companies/(MC)	46.10%***	44.92%***	39.43%	39.59%	8.72%	6.12%	8.77%	19.69%
% Amount to 10 largest awards companies/(MC)	54.59%***	57.35%***	52.21%***	51.88%	15.21%	10.94%	16.50%	27.16%

***. 5% significance value of being an Outlier (MAD)

Source: Own elaboration based on PCSP (2018-2021)

The analysis has not found excesses in the amount thresholds established by law to be issued as minor contracts, except for few cases in the four deputations. One relevant detail of the study of minor contracts is that several contracts, mostly of a public works nature, are very close to the 40,000 € threshold in the Deputation of Ourense. Contracts awarded to the same supplier in the same year for the same amount for different work items are also observed. Thus, it is common to find contracts for 39,669.42 € with different concepts, such as "rehabilitation of public service buildings"; "paving or improvement of infrastructures". In most cases, the difference in the object of the contract arises from the municipality in which the work is undertaken. However, there are cases where activities of very similar objects are carried out in the same municipality. Concerning equal award amounts from several suppliers, a clear example is that 24 different suppliers were granted 39,669.42 € in 102 contracts in 2020. This is consistent with Belmonte and Cabo (2020b), where contracts are awarded just at the limit of the thresholds and, on several occasions, to the same contractor. However, they also warn that not all contracts awarded in this way must be considered illegal. It would be necessary to examine the expedients to know whether they were for the same object.

To summarize, a set of indicators used show the concentration of awardees and resources in minor contracts. It should be noted the number of contracts with a value close to the threshold, and this trend increases during the pandemic. The results show a higher corruption risk in those deputations that mostly (over)use this type of public procurement. The higher spending executed through minor contracts and simplified processes is consistent with Rose-Ackerman (2021) research. Overall, the results suggest that the pandemic has favoured the use of minor contracts and a greater concentration of suppliers and funds. This can lead to an increase in the corruption risk at the local public procurement. In addition, a lack of transparency and the speeding of procedures hinders an effective control.

## Conclusions

Corruption at the local level is especially relevant, due to its direct effect on the local economy and closeness to citizens. As it is difficult to measure corruption at the local level, corruption risk is considered as a variable for approaching corruption at this level. The corruption risk does not inexorably imply corruption, but it does increase the probability that this event occurs. It should be noted that corruption risk has an impact on economies, because it derives from issues such as lack of confidence in the system, less transparency, and lower economic efficiency.

This paper deals with the corruption risk in local public procurement and the potential increase of this risk during pandemic. The Covid emergency results in speeding and loosening of procedures, which undermines transparency and hinders control.

This research focuses on minor contracts, a common type of public procurement, which allows circumvent ordinary procedures. Minor contracts avoid several formal issues, involving less transparency, publicity, and competition. This increases the opportunity for the misuse of public funds and enlarges corruption risk, leading to economic inefficiency. In addition to the inherent characteristics of minor contracts, the pandemic situation intensifies the risk.

The misuse of minor contracts often includes contracts just under the threshold or contracts awarded separately to the same contractor for recurrent actions or the same object, which suggests unduly splitting. The awarding of several contracts could also be due to poor procurement planning.

To analyse the corruption risk during the pandemic, this paper uses the case of the deputations, an intermediate level of government in Spain (NUTS 3). The deputations present peculiar characteristics that make them worthy of study. Thus, they act subsidiarily to entrust the provision of services in municipalities in a framework of limited and diffuse competences. They face difficulties for accountability given their lack of own revenue-raising capacity and that their government is not directly elected by citizens. These problems for accountability can also increase corruption risk in the local governments. Four deputations in the same regional context (Galicia) are selected because they show heterogeneity of responses to the pandemic and spending instruments at the local level, which enriches this contribution.

To measure the corruption risk in local public procurement, this paper proposes several indicators of concentration concerning minor contracts, such as of the number of contracts, amount, and suppliers. The link between concentration and higher corruption risk is suggested by literature (Heeks, 2011; Søreide, 2002; Telles, 2017).

The empirical analysis shows that deputations budgets have increased during 2020, due to the pandemic shock. However, the execution of spending shows different patterns among the four deputations analysed. Those deputations, which have traditionally allocated the most resources through minor contracts (Ourense and Lugo), have increased their use of this kind of public procurement. They present a higher corruption risk, as the different indicators of concentration suggest. The minor contracts for public works are overused by the Deputation of Ourense during 2020. Moreover, the number of contracts just at the thresholds has increased, as well as concentrated on a few suppliers and even on the same contractor on several occasions.

Overall, the study of the indicators regarding the concentration of minor contracts in the four deputations shows a higher concentration during pandemic both in resources and suppliers. This trend suggests a higher corruption risk in a singular context when public funds have increased, and the procedures have loosened.

This research contributes to the existing literature about corruption risk at the local level focusing on minor contracts. In addition, it analyses the effect of the pandemic, providing evidence of different response patterns by local governments. This heterogeneity leads to a differentiated impact on corruption risk. The context poses new challenges for local public procurement, which calls for a new framework to ensure transparency and efficiency, as Anessi-Pessina et al. (2020) underline. This case study uses the MAD methodology to identify structural changes, because of the limited available information. This methodology is applicable to other studies where data are limited.

Based on the analysis carried out, this paper proposes some recommendations. Local public procurement should be carried out through procedures that ensure transparency and competition, in line with the Consello de Contas (2018). Therefore, the urgency derived from the pandemic should not undermine the principles of transparency, competition, and efficiency. The use of minor contracts should be limited to strictly necessary cases, being an exception rather than the ordinary. Another recommendation is to better manage recurring and expected expenses, including the anticipation and the creation of a charter of suppliers for goods and services provided. A proper planning would save resources and increase transparency and competition of procurement (De Simone et al., 2017). Moreover, effective controls and measures to detect and prevent bad practices shall be designed and implemented (Lederman et al., 2005).

The main limitations of the study lie in the available data and the lack of homogeneity. Another limitation is the short time horizon since the outbreak of the pandemic emergency. This does not allow to assess whether the perceived changes are structural or arise from the current circumstances.

The future extensions of this research aim to delve deeper into the approach of a corruption risk indicator through factor analysis. It would also be interesting to widen the study for all Spanish deputations. Another interesting question is to study the association between the corruption risk and the cases of corruption in court or how the risk of corruption affects local and regional development.
